# Secondary somatosensory and posterior insular cortices: a somatomotor hub for object prehension and manipulation movements

**DOI:** 10.3389/fnint.2024.1346968

**Published:** 2024-04-25

**Authors:** Hiroaki Ishida, Laura Clara Grandi, Luca Fornia

**Affiliations:** ^1^Department of Neuroscience, Unit of Physiology, Parma University, Parma, Italy; ^2^Italian Institute of Technology (IIT), Brain Center for Social and Motor Cognition (BCSMC), Parma, Italy

**Keywords:** hand manipulation, somatomotor integration, secondary somatosensory cortex, insular cortex, attention, lateral grasping network

## Abstract

The secondary somatosensory cortex (SII) and posterior insular cortex (pIC) are recognized for processing touch and movement information during hand manipulation in humans and non-human primates. However, their involvement in three-dimensional (3D) object manipulation remains unclear. To investigate neural activity related to hand manipulation in the SII/pIC, we trained two macaque monkeys to grasp three objects (a cone, a plate, and a ring) and engage in visual fixation on the object. Our results revealed that 19.4% (*n* = 50/257) of the task-related neurons in SII/pIC were active during hand manipulations, but did not respond to passive somatosensory stimuli. Among these neurons, 44% fired before hand-object contact (reaching to grasping neurons), 30% maintained tonic activity after contact (holding neurons), and 26% showed continuous discharge before and after contact (non-selective neurons). Object grasping-selectivity varied and was weak among these neurons, with only 24% responding to fixation of a 3D object (visuo-motor neurons). Even neurons unresponsive to passive visual stimuli showed responses to set-related activity before the onset of movement (42%, *n* = 21/50). Our findings suggest that somatomotor integration within SII/pIC is probably integral to all prehension sequences, including reaching, grasping, and object manipulation movements. Moreover, the existence of a set-related activity within SII/pIC may play a role in directing somatomotor attention during object prehension-manipulation in the absence of vision. Overall, SII/pIC may play a role as a somatomotor hub within the lateral grasping network that supports the generation of intentional hand actions based on haptic information.

## Introduction

The secondary somatosensory cortex (SII) and the adjacent posterior insular cortex (pIC) play a fundamental role in high-level somatosensory processing such as object identification and tactile learning (Mishkin, 1979; Murray and Mishkin, 1984; Reed and Caselli, 1996). In both human and non-human primates, the SII/pIC exhibits multiple representations for fingers and hands (Robinson and Burton, 1980; Krubitzer et al., 1995; Fitzgerald et al., 2004; Eickhoff et al., 2007; Vecchio et al., 2021), and convergent results from classical hodological studies in non-human primates demonstrated the connections between hand regions within SII/pIC and parieto-premotor hand-manipulation-related areas, such as the ventral premotor area F5 and the anterior intraparietal area AIP (Matelli et al., 1986; Borra et al., 2008; Gharbawie et al., 2011). The F5-AIP pathway is the core of the so-called lateral grasping network (Borra et al., 2017) playing a crucial role in the visuomotor transformation necessary to grasp objects, and it is considered a cognitive interface for hand actions (Borra and Luppino, 2018). The visuomotor model for object grasping proposes that area AIP transmits visual information about three-dimensional (3D) objects to area F5 to select the pattern of hand movement, while area F5 sends back the motor signal (efference copy) of the chosen motor command to area AIP (Jeannerod et al., 1995; Sakata et al., 1997; Fagg and Arbib, 1998).

To clarify the involvement of SII/pIC within the lateral grasping network, in our previous research, we recorded single neurons in the SII/pIC hand region that selectively respond to grasping according to the type of manipulation act rather than the type of object shape. Importantly, these neurons did not have passive somatosensory properties (Ishida et al., 2013). However, that research did not investigate grasping movements towards 3D objects of different shapes, leaving questions unanswered regarding the selectivity of SII/pIC neurons for object manipulation, observation, and their potential involvement in the predictive control of motor commands when compared to the functional hypothesis of areas F5-AIP (Murata et al., 1997, 2000).

To address these questions, our current study involved training monkeys to perform hand manipulation tasks using three different objects (a cone, a plate, and a ring). We hypothesized that activation of SII/pIC neurons during object prehension-manipulation would reflect the direct or indirect influence of selected motor signals or the predicted sensory consequences of motor commands, reflecting its role as the somatomotor hub within the lateral grasping network. In terms of visual responses, we expected that neurons in these areas might be involved in directing somatomotor attention during object prehension-manipulation, rather than encoding object visual features (Hsiao et al., 1993; Burton and Sinclair, 2000; Romo et al., 2002), possibly reflecting connections with prefrontal areas involved in executive functions (Borra and Luppino, 2018).

## Materials and methods

### Subjects, training, and surgical procedures

Two male macaques (*Macaca mulatta*) were used in this study. We recorded the right hemisphere in one monkey (MK2, 4.5 kg) and the left hemisphere in another monkey (MK3, 5.0 kg). Before recording, each monkey was accustomed to sitting comfortably in a primate chair, interacting with the experimenters, and familiarizing themselves with the experimental setup. They were trained to perform the hand manipulation task described below, using the hand contralateral to the recorded hemisphere. At the end of training, a head fixation system (Crist Instrument, Hagerstown MD, United States) and a cylindrical-recording chamber (Narishige, Tokyo, Japan, inner diameter = 20 mm) were implanted under general anesthesia (ketamine hydrocloride, 5 mg/Kg, i.m. and medetomidine hydrocloride 0.1 mg/Kg i.m.), followed by post-surgical pain medications. The position of chambers allowed recording from the rostral to the middle part of the upper bank of the lateral sulcus, including the hand regions of SII/pIC (Ishida et al., 2013; Grandi and Gerbella, 2016).

All experimental protocols were approved by the Ethics Committee for Animal Research of the University of Parma and the Superior Institute for Health. The authorization to conduct our experiments was provided by the Division of Animal Health and Veterinary Medication of the Department of Public Veterinary Health, Nutrition and Food Safety of the Italian Ministry of Health.

## Behavioral paradigms

### Clinical examinations

Isolated neurons were first investigated with clinical examinations to distinguish between motor neurons (those responding only to active hand movements) and somatosensory neurons (those exhibiting a clear tactile and/or proprioceptive receptive field; RF). Hand-related motor activity was tested during a food grasping task, where monkeys were given the opportunity to pick up 1 cm cubes of potato; similarly, mouth-related activity was tested during oral and alimentary movements. To determine the presence or absence of somatosensory RFs in which neurons respond to hand movements, the experimenter directly manipulated the monkeys’ upper limbs, hands, and fingers (details in the Supplementary information file). Based on the results of clinical examinations, we classified neurons as tactile and proprioceptive (responding to passive somatosensory stimuli) or hand-related motor neurons (nonresponding to passive somatosensory stimuli). The clinically isolated units were then more carefully investigated with hand-manipulation-controlled tasks (see below).

### Hand manipulation and control tasks

**Figure 1:**
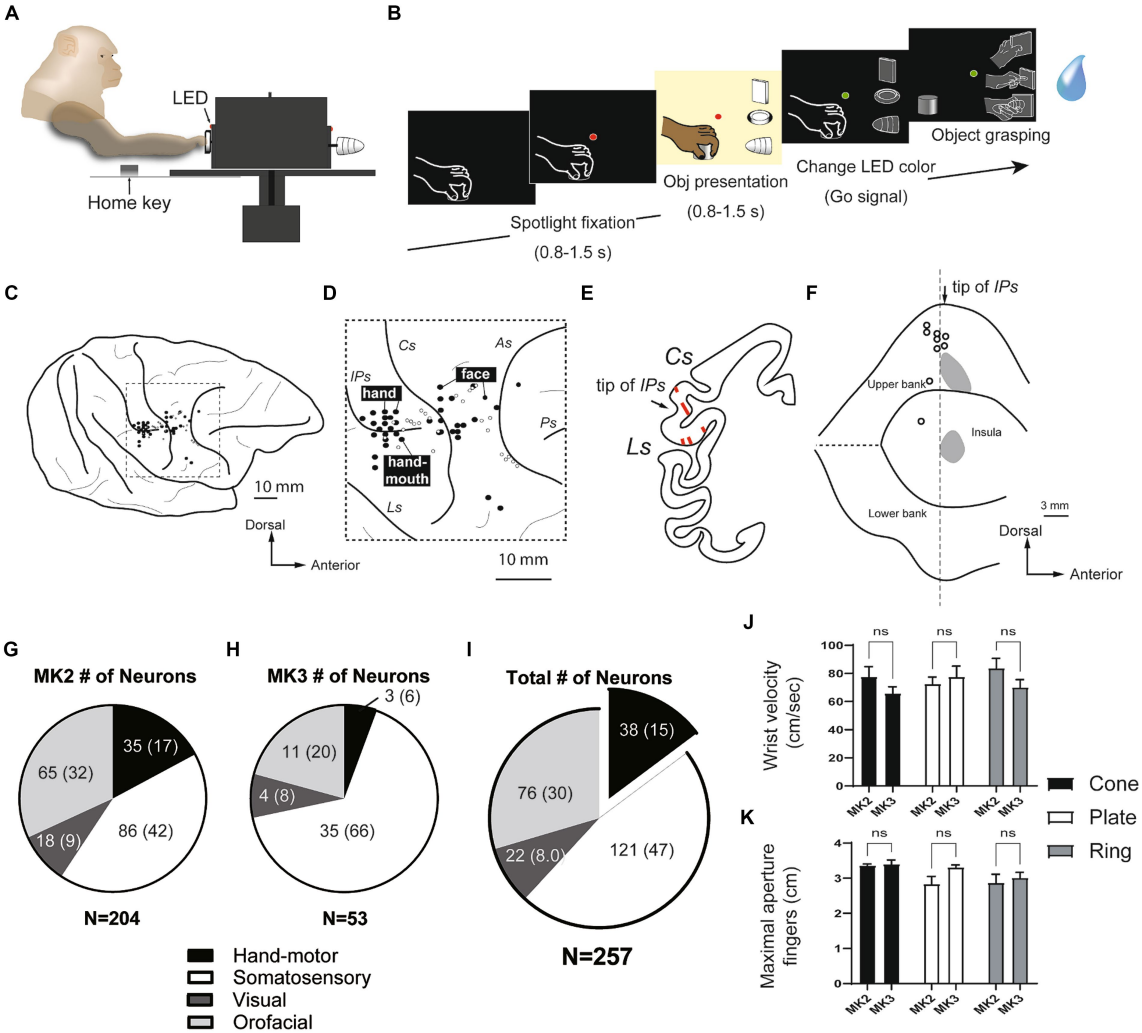
Hand manipulation task and characteristics of neurons recorded within the SII hand region and the posterior insular cortex (pIC). **(A,B)** Hand manipulation task: Side view of the task apparatus and the task sequence. **(C,D)** Recording sites: Body representations (black filled circles) on the cortical surface surrounding the right hemispheric interparietal sulcus (IPs) and the central sulcus (Cs) in MK2. Hand, finger, and mouth representations were recorded adjacent to the anterior apical part of the IPs. The more anterior region around the Cs appeared to be associated with facial representations. The white circles indicate the locations of the lesion tracks, which represent the microelectrode penetrations, and were reconstructed from Nissl sections. **(E,F)** Reconstructed recording sites: **(E)** An example of a Nissl section displaying the SII hand region. Arrows indicate the position of the tip of the IPs, and lines within the areas represent penetration tracks. **(F)** A map of the lateral sulcus unfolds, with gray areas denoting the recording sites of the SII hand region on the upper bank of the lateral sulcus and the pIC. These sites are determined on the basis of lesion tracks that indicate microelectrode penetrations in the Nissl section. White circles correspond to the locations of the lesion tracks, as illustrated in panel **(D)**. IPs, intraparietal sulcus; Cs, central sulcus; Ls, lateral sulcus; As, arcuate sulcus; Ps, principal sulcus. **(G–I)** Characteristics of the single cells recorded from two monkeys. A subset of the recorded neurons (*n* = 38, 15%) were identified as hand motor neurons, exclusively involved in hand manipulation; however, they did not show somatosensory receptive fields (proprioceptive and superficial tactile RFs). **(J,K)** Kinematics of grasping objects (cone, plate, ring). The speed of reaching the object (wrist velocity in cm/sec) and the maximum aperture between the tips of the thumb and index finger (in cm) showed no significant differences, not only between two monkeys, but also between three objects.

The hand manipulation task was a modified version of a paradigm originally devised by Sakata and coworkers (Figures 1A,B; Murata et al., 1997, 2000; Raos et al., 2006). The monkey was seated in a primate chair in front of a turntable, separated into three sectors, each containing a solid of different shapes: cone, plate, and ring. The task (Figure 1B) started when the monkey held its hand in a fixed starting position (Home key), after a variable intertrial period ranging from 1 to 1.5 s. When the red LED is on, the monkey was trained to fixate on it for 0.5–0.8 s (Spotlight fixation). At the same time, an ambient light is turned on to illuminate the object to be grasped for 0.5–0.8 s (Object presentation). When the color of the LED changed from red to green (Go signal), the illumination light was automatically turned off. The monkey was trained to perform subsequent motor acts in complete darkness (Object grasping): release the home key, reach it forward, grasp the object, and then pull it within 1.2 s. The object had to hold steadily for at least 0.8 s. If the task was performed correctly, the reward was automatically delivered (Pressure reward delivery system, Crist Instruments). We recorded 15–18 trials for each condition.

In all sessions, we also recorded three control tasks: (1) spotlight fixation task. To determine whether the neuron responded to the spotlight LED and the movements of the mouth and tongue to obtain reward juices, the monkey was completely in darkness and with its hand in the home key position, with the fixation point on; after a variable time lag (< 1.0 s) from the onset of the fixation, the reward was delivered; (2) object fixation task. To determine whether the neuron responded to the sight of the object, the monkey was trained to fixate the object without manipulating it. When the green LED was turned on, it was associated with object fixation trials. After the monkey fixed on it by holding a home key for 1.0 s, the ambient light is turned on to illuminate the object, and the monkey had to stay fixed for 0.5–0.8 s; (3) air puff stimulation. On the basis of the result of our clinical examination, if we found somatosensory receptive fields (RFs) on the fingers, we applied an air puff stimulation (Air puff delivery system, Crist Instruments) at a consistent pressure of 0.1 bar (Avillac et al., 2005). After the monkey fixated on a green LED, air puff stimulation was provided towards the finger (< 0.5 s). We recorded 15–18 trials for control tasks.

### Unit recording and data analysis

Single unit recoding was performed using a 16 channel Omniplex recording system (Plexon Inc., Dallas, TX, United States) with a 16 channel V-probe. Online spike sorting was performed on all channels using dedicated software (Plexon), but all final quantitative analyses were performed offline. The experiment was controlled by LabView-based software. The digital signals provided time-related information about each task epoch (ex. the onset and offset of fixation point) and the behavioral event (e.g., monkey hand contact with the home key). The position of the eyes was monitored in parallel with neuronal activity with an eye tracking system composed of a camera (Iscan Inc., Cambridge, MA, United States, ETL-200). Eye position signal was monitored by the same LabView-based software dedicated to the control of the behavioral paradigm.

Based on digital signals related to the main behavioral events (Figure 1B), we defined different epochs of interest of the hand manipulation task: (1) baseline, from 500 ms before object presentation to this event; (2) pre-contact, from 500 ms before hand-object contact onset to this event; (3) post-contact, from hand-object contact onset to 500 ms after this event. We defined epochs in the spotlight fixation task: (1) baseline, from 500 ms before turning the LED on; (2) fixation, from the LED onset to 500 ms after this event. As to object fixation task: (1) baseline, from 800 ms before turning the LED; (2) object fixation, from the light on onset to 800 ms after this event. We defined epochs in air puff stimulation: (1) baseline, from 500 ms before turning on the LED; (2) air puff stimulation, from air puff onset to 500 ms after this event.

The mean discharge frequency during the above-defined stimulation epochs for each task was compared with mean activity during baseline by means of a 3 × 3 repeated measures ANOVA (factors: Object, Epoch) following Tukey HSD post-hoc tests, a one-way repeated measures ANOVA (spotlight fixation task) and the *t*-test (object fixation and air puff stimulation tasks), respectively (*p* value <0.05). Statistical analyses were performed with Matlab (The MathWorks Inc., MA, United States) and Prism 10.0 (GraphPad).

Combining the results of the clinical examinations and the controlled tasks (hand manipulation task, spotlight fixation, object fixation, and air puff), the neurons were defined as:

1. Orofacial neurons: They responded to mastication during clinical examinations. Coherently, their activity was higher during reward-taking than during baseline in the spotlight fixation task.
2. Tactile neurons: Neurons showed a superficial tactile response on the skin surface of the upper limb, hand, and fingers during clinical examination.
3. Proprioceptive neurons: Neurons that responded during clinical examinations to passive displacement of the joint.
4. Hand motor neurons: Neurons that responded only to grasping food during clinical examinations. They exhibit higher neuronal activity during the hand manipulation task in the pre-and/or post-contact epochs than during baseline. Finally, they did not respond to object fixation.
5. Visuo-motor neurons: Neurons that responded to object fixation in addition to the criteria of hand motor neurons.
6. Visuo-somatosensory neurons: Neurons responded to object fixation in addition to the criteria of tactile and proprioceptive neurons.

To quantify the preference of recorded single neurons for the different grip types of objects, we calculated a preference index (PI) considering the magnitude of the neuronal response to the three grips (more details in the Supplementary information file).

The population response was calculated as a net normalized mean activity. First, the mean activity was calculated for each 20 msec bin through all recording trials for each condition. Then an offset procedure was applied for each condition, subtracting the mean baseline activity from the value of each bin (net activity). For each neuron, the peak discharge was found for all task conditions during task-related epochs and used to normalize the activity of each condition. To statistically compare responses in different populations, we used the net normalized mean activity as a dependent variable. We then performed a 3 × 2 repeated measures ANOVA (factor: 3 Object, 2 Epoch: pre-contact, post-contact) followed by Tukey HSD post-hoc tests (*p* < 0.05).

### Histological analysis

After the experiment was complete, a series of electrolytic lesions were performed, and stereotaxic penetration coordination was used to reconstruct the unit recording sites. The relative positions of the penetrations to the electrolytic lesion were then determined indirectly, and the sites were plotted on an unfolded map of the lateral sulcus (Ls, Figures 1C–F, more details in the Supplementary information file and Supplementary Figure S1).

## Results

### Multiple body representations in the SII/pIC

We recorded a total of 257 single units from the posterior inner perisylvian region, including hand-fingers region of SII and the adjacent region of the posterior insular cortex (pIC) of two macaque monkeys. Figures 1C–F shows examples of the anatomical location of the investigated regions in MK2. The MK2 was explored from the middle part of upper bank (SII), while MK3 was intensively investigated from the pIC (204 in the right hemisphere of MK2, 53 in the left hemisphere of MK3, Figures 1G,H).

Since the aim of the study was to investigate the SII/pIC neurons in the context of the lateral grasping network, our analysis focused on hand motor neurons (type 4) and visuo-motor neurons (type 5). The other categories were briefly summarized. Based on the result of our clinical examinations and the hand manipulation tasks, 159 of 257 neurons (62%) were categorized as hand-related motor neurons (*n* = 38, 15%) and somatosensory (*n* = 121, 47%) (Figure 1I). The somatosensory neuron showed clear somatosensory RFs on the upper limb, hand and fingers (tactile neurons, n = 80; proprioceptive neuron, *n* = 41), while hand-related motor neurons did not. Twenty-two of 257 neurons (8%) showed responses during both object fixation and hand manipulation (visuo-motor/somatosensory neurons). Twelve of them did not show clear somatosensory RFs (visuo-motor neurons), and 10 neurons showed somatosensory RF on the hands and arms (visuo-somatosensory neurons). Seventy-six of 257 neurons (30%) were mouth-related somatosensory or motor neurons. Through the two monkeys, pIC showed more neurons that did not respond to the hand manipulation task compared to SII.

### Three types of hand-manipulation-related neurons

Kinematics analyses were performed on both monkeys to confirm the presence of potential differences in the parameters of hand movement while performing the hand manipulation task. The results did not show significant differences between the three objects or between the two monkeys (Figures 1J,K; Supplemental information).

**Figure 2:**
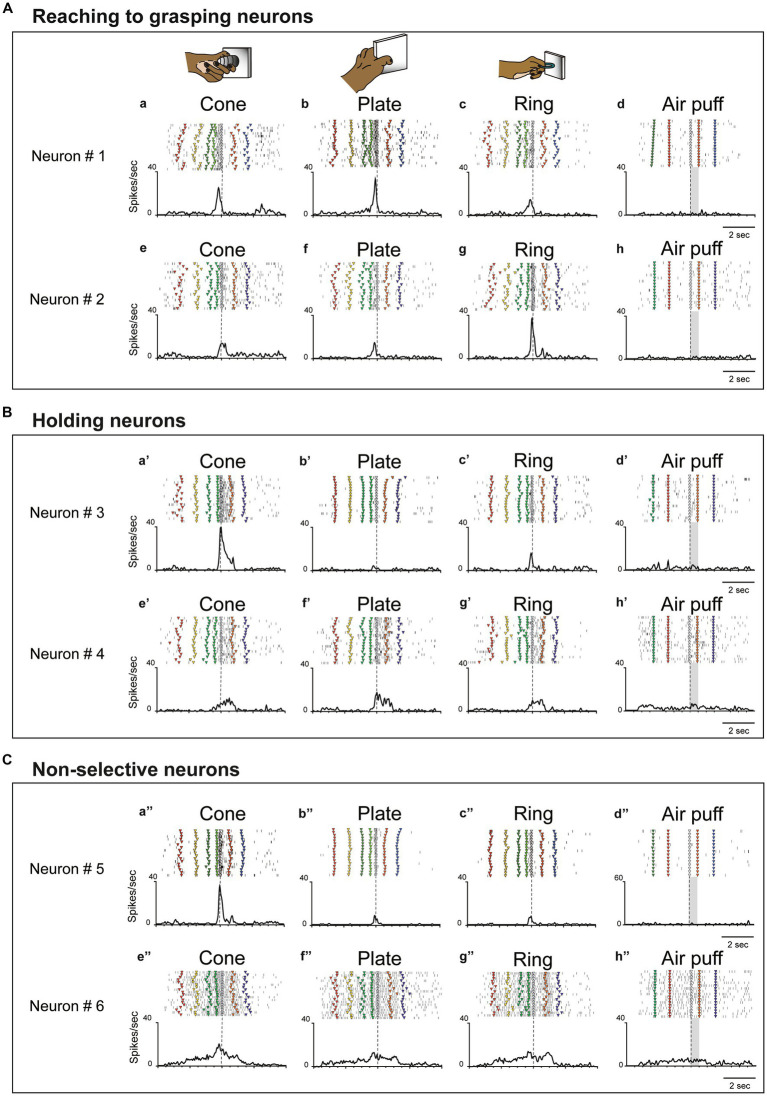
Examples of three types of hand-manipulation-related neurons. **(A)** Neurons #1 and #2 are examples of reaching to grasping neurons demonstrating stronger responses during the pre-contact epoch compared to the post-contact epoch. Task-related neural activity is observed for the (a,e) cone, (b,f) plate, and (c,g) ring. The object selectivity, measured as the preference index (PI), is moderate for these neurons (PIs = 0.26 and 0.39, respectively). In panels (a–c,e–g), the dotted line indicates the timing of the monkey’s contact with the object. (d,h) These neurons did not possess a somatosensory receptive field and did not induce responses to air puff stimulation. Dotted lines mark the onset of the stimulus, while gray rectangles represent the duration of the stimulus. The markers within each raster depict the behavior of monkeys during task execution. The behavioral labels for each marker are as follows: red inverted triangles denote the initiation of spotlight fixation; yellow indicates object presentation; green indicates the go signal; yellow green indicates movement initiation; gray indicates object contact; orange indicates object release, and blue indicates reward delivery. **(B)** Neurons #3 and #4 are examples of holding neurons that demonstrate stronger responses during the post-contact epoch compared to the pre-contact epoch (a’–c’,e’–g’). The object selectivity for these neurons is measured as PIs = 0.80 and 0.29, respectively. In panels (d’,h’), these neurons do not respond to stimulation by air puff. The meaning of markers and other elements on the raster is described above. **(C)** Neurons #5 and #6 are examples of non-selective neurons which did not show significant difference between pre-contact and post-contact epochs (a”–c,”e”–g”). The object selectivity for these neurons is measured as PIs = 0.80 and 0.23, respectively. In panels (d”,h”), these neurons do not respond to air puff stimulation. The meaning of markers and other elements on the raster is described above.

To clarify action selectivity (from reaching grasping to holding) and object-selectivity, we analysed 50 neurons related to hand manipulation, including hand motor neurons (*n* = 38) and hand-visuo-motor neurons (*n* = 12). Figures 2A–C show representative examples of neurons related to hand manipulation. All hand manipulation-related neurons lacked a somatosensory receptive field and did not show a significant response to air puff stimulation (see Figures 2A–d, B–d’, C–d”).

**Figure 3:**
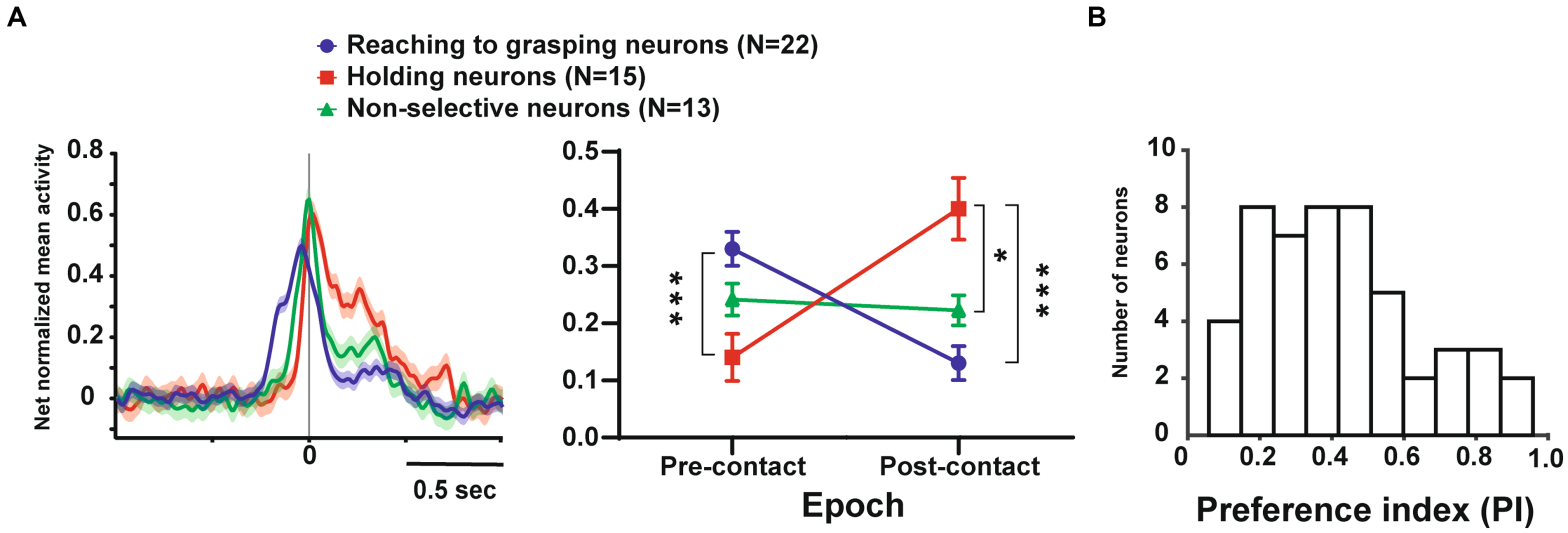
Temporal profile of neuronal activity of the population and selectivity of objects in these neurons. **(A)**. (Left) Temporal profile of the mean normalized activity of the entire neuronal population for each type. Neuronal activity is aligned with the monkey’s hand-object contact. (Right) Mean net normalized response in reaching to grasping, holding, and non-selective neurons in pre-and post-contact epochs. The bar indicates the standard error of the mean (SEM), with * indicating *p* < 0.05. **(B)**. Distribution of the PI values for all neurons (*n* = 50).

Since single unit analysis demonstrated a significant difference between pre- and post-contact activity, we further investigated the temporal profile of their discharge. We statistically subdivided hand-manipulation-related neurons into 3 types: (1) reaching to grasping neurons (*n* = 22/50), which showed significantly stronger responses in the pre-contact epoch than in post-contact one (ex. Figure 2A, neurons #1, #2); (2) holding neurons (*n* = 15/50), which showed significantly stronger responses in the post-contact epoch than in the pre-contact one (ex. Figure 2B, neurons #3, #4); (3) Non-selective neurons (*n* = 13/50), which did not show significant difference between the two epochs (ex. Figure 2C, neurons #5, #6). Figure 3A shows the relationship between hand-manipulation-related activity and the temporal discharge profile of the three neuronal populations. As mentioned above, reaching to grasping neurons showed significantly stronger activity in pre-contact epoch (*p* < 0.0001), holding neurons showed significantly stronger activity in post-contact epoch (*p* < 0.0001) and non-selective-neurons did not show any significant difference between the two epochs.

In terms of object selectivity, single neurons exhibited significantly stronger responses during hand manipulation involving one or more objects. For example, neurons #3 and #5 in Figures 2B,C responded only to the cone (PIs = 0.8, respectively) while neurons #1, #2, #4 and #6 in Figures 2A–C show a low selective response to specific objects (PIs = 0.26, 0.39, 0.29, 0.23, respectively). The median PI value of all neurons related to hand manipulation is 0.41 and approximately 75% of the total neurons (*n* = 39/50) showed PI values below 0.50 (Figure 3B). The percentages of the best objects that show the strongest response to grasping three objects in hand-manipulation-related neurons are the cone (*n* = 17, 34%), plate (*n* = 23, 46%), ring (*n* = 10, 20%). Furthermore, comparing the PI values between 3 populations of hand-manipulation-related neurons found that they did not show significant differences among the groups (mean PI: reaching to grasping, 0.38; holding, 0.48; non-selective, 0.42, *p* = 0.54).

### Object visual and set-related activity

**Figure 4:**
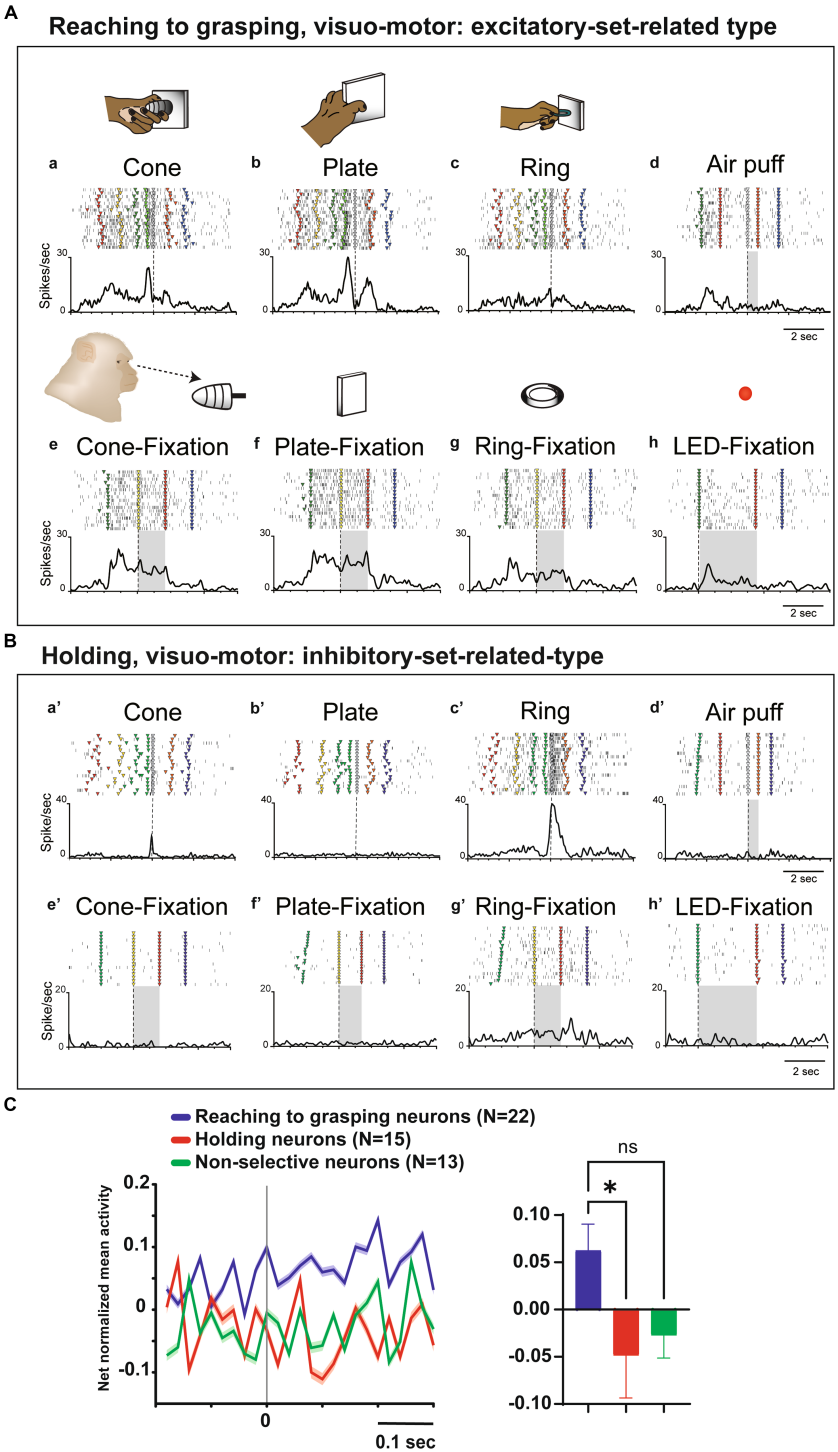
Visual and set-related activities of neurons related to hand manipulation. **(A)**. An example of a reaching-to-grasping type of hand-visuomotor neuron. Task-related neural activity is observed for the (a) cone, (b) plate, and (c) ring. The object selectivity for this neuron is measured as PI = 0.31. In panel (d), the neuron lacked a somatosensory receptive field and remained unresponsive to air puff stimulation. In panels (e–h), this neuron did exhibit significant visual responses during both object fixation and LED fixation tasks. This neuron shows significantly stronger set-related activity compared to baseline activity and is of the excitatory type. **(B)** An example of holding type of hand visuomotor neuron. Task-related neural activity is observed for the (a’) cone, (b’) plate, and (c’) ring. The object selectivity for this neuron is measured as PI = 0.95. In panel (d), the neuron lacked a somatosensory receptive field and remained unresponsive to air puff stimulation. In panels (e’-h’), this neuron exhibits significant visual responses during the fixation of the ring object. This neuron shows significantly weaker set-related activity compared to baseline activity and is of the inhibitory type. **(C)** (Left) Temporal profile of the mean normalized activity of set-related activity of the whole neuronal population for each type. Neuronal activity is aligned with the go signal. (Right) Mean net normalized response of set-related activity in 3 types. The bar indicates the standard error of the mean (SEM), with ^*^ indicating *p* < 0.05.

Twelve visuo-motor neurons lacked somatosensory RFs but exhibited significant responses during hand manipulation and object fixation for at least one of the three objects (visuo-motor neurons; Figures 4A,B). Figure 4A shows an example of reaching to grasping type of visuo-motor neuron. In the context of cone manipulation, the neuron exhibited significant activity only during the pre-contact epoch (Figure 4Aa). Conversely, for plate manipulation, this neuron demonstrated notable activity during both the pre-contact and post-contact epochs (Figure 4Ab) and the same neuron did not respond during ring manipulation (Figure 4Ac). The PI value of this neuron is 0.31. Importantly, the neuron presented substantial responses during fixation on all objects (Figures 4Ae–g), as well as during the fixation of the spotlight LED (Figure 4Ah). Figure 4B shows an example of a holding type of visuo-motor neuron. This neuron exhibited selective responses to ring manipulation only during the post-contact epoch; therefore, the PI value of this neuron is 0.95 (Figures 4Ba’–c’). The neuron showed significant responses during fixation to the ring object but did not respond to fixation to other objects (Figures 4Be’–g’) and the spotlight LED (Figure 4Bh’).

To clarify the relationship between visual information and hand manipulation, we further investigate activity during the set-related period, the transition period between the object presentation and motor initiation; from 200 ms before to 300 ms after the Go signal (*cf.* Murata et al., 1997). Among the 12 hand-visuo-motor neurons, 7 neurons (58.3%) showed significant excitatory or inhibitory activity during the set-period. Although none of the hand motor neurons (*n* = 38) showed significant responses during the object fixation control task, 14 of 38 (36.8%) neurons showed significant activity during the set-related period. In a population analysis of set-related activity, the net normalized mean activity of the reaching to grasping neurons were significantly higher than that of the holding neurons (*p* = 0.03, Figure 4C).

## Discussion

The purpose of this study was to elucidate the functional roles related to grasping 3D objects in the monkey SII/pIC hand-manipulation-related neurons. In this preliminary study, our aim was to characterize the functional roles of SII/pIC as a somatomotor hub region within the lateral grasping network using the task device and sequence developed by Sakata and his coworkers for 3D object hand manipulation (Jeannerod et al., 1995; Murata et al., 1997, 2000; Sakata et al., 1997; Raos et al., 2006).

We found that 19.4% of the recorded SII/pIC neurons (*n* = 50/257) were hand-manipulation-related. These neurons did not exhibit passive somatosensory responses on the hand and fingers, but showed vivid discharges only when monkeys grasped an object. When examining the temporal profiles of their neural activity, we classified hand-manipulation-related neurons into three distinct motor neuron types: reaching to grasping, holding, and non-selective neurons (Figures 2, 3A). These properties are consistent with the findings of our previous study (Ishida et al., 2013); namely, reaching to grasping neurons displayed discharges before hand-object contact, while non-selective neurons also exhibited higher firing rates before hand-object contact, albeit peaking at the time of contact. We propose that the activity of both reaching to grasping and non-selective neurons may reflect efference copy or corollary discharge of selected motor commands from PMv, allowing for sensory prediction and optimization of motor control (Wolpert and Ghahramani, 2000; Brown et al., 2011; Limanowski et al., 2019). Moreover, SII/pIC shows anatomical connection with the superior dorso-medial parietal areas (Disbrow et al., 2003; Bakola et al., 2010) and caudal inferior parietal area PG (Rozzi et al., 2006). This evidence suggests that SII/pIC may functionally interact directly, or indirectly through PG, with the medial grasping network, involving parietal area (V6A) and the dorsal premotor area (F2) (Galletti et al., 2003; Breveglieri et al., 2016; Filippini et al., 2017), thus possibly playing an extended sensorimotor control that subserves all phases of prehension actions and not only holding and manipulation.

In contrast, holding neurons demonstrated a significant increase in activity immediately after contact with the hand-object. Generally, hand-object contact information serves as an essential tactile cue to timing the sequence of hand-object interaction (Hikosaka et al., 1985; Johansson and Flanagan, 2009) and to adjust current or memorized motor commands for dexterous hand manipulation (Jenmalm and Johansson, 1997). Holding neurons receive information related to these sensorimotor processes and are believed to maintain the required task-related muscle activity to hold the grasped object (Macefield et al., 1996).

A significant portion of neurons associated with hand manipulation in SII/pIC did not exhibit responses to visual stimuli of 3D objects. In particular, visuomotor neurons, as documented in the AIP region of macaque monkeys by Murata et al. (2000), that is, neurons displaying a preference for motion and synchronized visual responses to objects (see Figures 4A,B), may be scarce within the hand region of SII/pIC, despite the presence of robust reciprocal anatomical connections. Along this line of results, the object selectivity of SII/pIC was not pronounced, as many neurons responded to one or more object grasping tasks. The same applies to the results of the population analysis; the PI did not show significant differences among the three types of motor neurons (see Figures 2, 3B). In recent single-unit studies with monkeys, SII was observed to respond to a range of visual stimuli, such as peri-personal space stimulation, active hand movements, and observation of reaching and grasping actions (Taoka et al., 2013; Hihara et al., 2015). These findings align with both monkey and human functional magnetic resonance imaging studies, which indicate the involvement of SII and the pIC when observing another individual’s body being touched (Keysers et al., 2004). These results imply that SII and pIC might play a role in processing socio-cognitive relevant information rather than 3D object.

Even neurons that are not responsive to passive visual stimuli displayed responses during the set-related period both before and after the initiation of movement, followed by the presentation of the object (Soso and Fetz, 1980; Kurata and Wise, 1988; Nelson, 1988). Interestingly, among these neurons, reaching to grasping neurons were primarily excitatory, whereas holding neurons exhibited inhibitory responses. It is well accepted that the activity of SII neurons in macaque monkeys may be influenced by attentional modulation during task behavior (Hyvarinen et al., 1980; Hsiao et al., 1993; Burton and Sinclair, 2000). This includes neurons responding when the monkey focuses on fingertip contact with an object, neurons that fire prior to contact, and neurons that show further inhibition (Iriki et al., 1996). These properties may be consistent with reciprocal connections with prefrontal areas within the lateral grasping network (Borra and Luppino, 2018), which may be involved in tuning the SII/pIC firing rate based on attentional processes, finally contributing to executive control of hand actions. In our previous study (Ishida et al., 2013), we showed that the responses of hand-manipulation-related neurons in the SII/pIC were enhanced in a dark environment without visual information about the grasped target. Collectively, these neurons in this area may play a role in directing somatomotor attention during hand manipulation in the absence of vision. This potential involvement suggests a role in the predictive sensory outcomes from motor commands and a potential contribution to dexterous hand manipulation (also Supplementary information; Supplementary Figure S2).

## Conclusion

The present findings suggest that both the hand-manipulation-related and somatosensory-related neuronal populations probably contribute to somatomotor processing from the initial to the final phase of object prehension and manipulation. We propose that activation of SII/pIC neurons during hand manipulation involves the direct or indirect influence of selected motor signals or the prediction of sensory outcomes from motor commands. This hypothesis may support the role of SII/pIC regions as a somatomotor hub within the lateral grasping network.

## Data availability statement

The raw data supporting the conclusions of this article will be made available by the authors, without undue reservation.

## Ethics statement

The animal study was approved by the Ethics Committee for Animal Research of the University of Parma and the Superior Institute for Health (appraisal No. 2783, 26/01/2010). The study was conducted in accordance with the local legislation and institutional requirements.

## Author contributions

HI: Conceptualization, Data curation, Formal analysis, Funding acquisition, Investigation, Methodology, Project administration, Supervision, Validation, Visualization, Writing – original draft, Writing – review & editing. LG: Data curation, Formal analysis, Investigation, Writing – review & editing, Project administration. LF: Conceptualization, Data curation, Formal analysis, Methodology, Writing – review & editing, Validation.
