# Supplementary material for: Secondary somatosensory and posterior insular cortices: a somatomotor hub for object prehension and manipulation movements
Source: Front Integr Neurosci. 2024 Apr 25;18:1346968. doi: 10.3389/fnint.2024.1346968 (PMC11079213; doi:10.3389/fnint.2024.1346968)
Supplement: Supplementary file 1 [file Data_Sheet_1.PDF]

Supplemental information

Clinical examinations of somatosensory neurons

Passive somatosensory stimuli consisted of a) ‘tactile’ stimulation, i.e. hair deflection by touch or light and deep pressure to stimulate different types of skin receptors; b) ‘proprioceptive’ stimulation, i.e. slow and fast passive joints movement of the upper limb (the shoulder, elbow and wrist) and fingers phalanxes. Neuronal activity was observed while passive somatosensory stimuli were applied to the body parts of the monkeys using the experimenter's hand in the absence of visual feedback. If we found superficial tactile RFs, we applied a stimulus to the body part. If we found dominant proprioceptive responses, we manipulated the joint (ex. flexion or extension).

Preference index (PI)

To quantify the preference of recorded single neurons for the different grip types of objects, we calculated a preference index (PI) considering the magnitude of the neuronal response to the three objects. It was calculated as follows:

$$PI = \frac{n - \left(\frac{\sum r_i}{r_{\max}} \right)}{n-1},$$

where n is the number of objects ($n = 3$), r_i is the mean firing rate of the neuron in its pre- and post-contact epoch of each object and $r_{\max}$ is the maximal mean value for the preferred object during its pre- and post-contact epochs. PI values can range from 0 (the discharge is identical among objects) to 1 (maximal selectivity for one object).

Histological analysis

To accurately identify the location of the recording sites, at the end of the recording sessions and 10 days before sacrificing the monkey, electrolytic lesions (10 μA cathodic pulses, duration 10 s) were performed in both hemispheres. In detail, for each hemisphere, known coordinates of the external borders of the recorded regions were selected, and in each of them four lesions were made at different depths. The monkey was then deeply anesthetized with an overdose of sodium thiopental and consecutively perfused with saline, 3.5–4 % paraformaldehyde, and 5 % glycerol, prepared in 0.1 M phosphate buffer and pH 7.4, through the left cardiac ventricle. The brain was then coronally blocked in a stereotaxic, removed from the skull, photographed, and placed in 10 % buffered glycerol for 3 days and 20 % buffered glycerol for 4 days. Finally, it was frozen coronal sections of 60 μm thickness, and the obtained sections will be processed with Nissl staining (0.1 % thionin in 0.1 M acetate buffer, pH 3.7) to identify electrolytic lesions (Fig.S1). The 2D reconstruction of the upper bank of the lateral sulcus and of the posterior insula was aligned along its fundus (for more information, see supplementary information (S1) in Ishida et al.2013).

Fig. S1

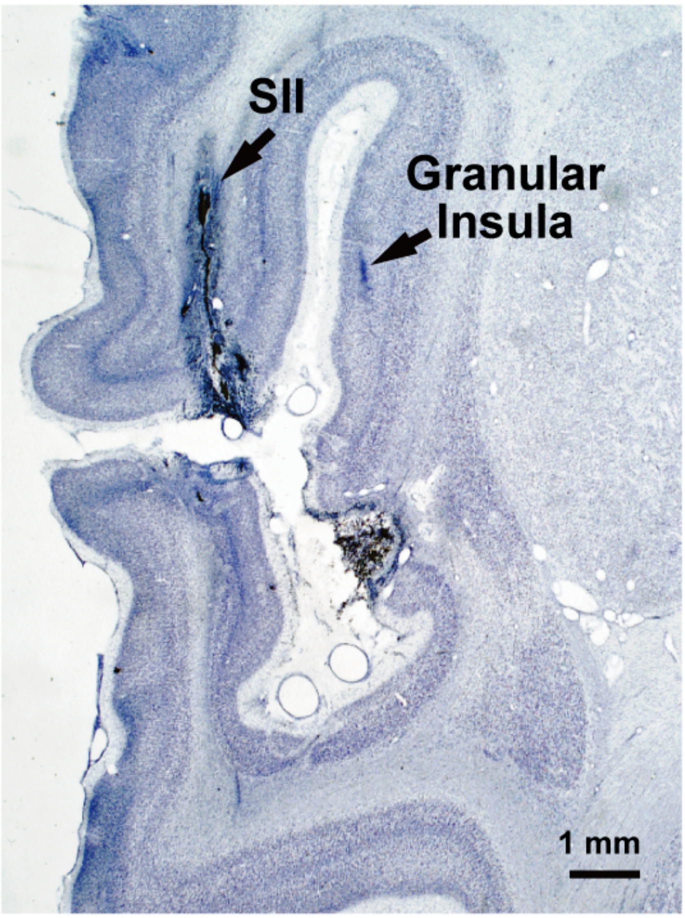

Figure S1: Example of a Nissl section showing the MK2 recording site
The black arrows in the photograph indicate the lesion tracks in the SII cortex and the insular cortex.

Kinematic analyses

We focused on 1) reaching and pre-shaping time as the wrist velocity (cm/sec) and 2) maximal aperture fingers (cm) during the execution of each object condition. White markers were placed on the styloid process part of the radius, the tip of the last phalanx of the thumb, and the index finger of the monkey. By means of a digital video camera, we captured each hand-manipulation, and analysed it off-line by means of dedicated software, at a sampling rate of 25 frames/second. For each kinematic index, a 2 x 3 repeated measures ANOVA with monkey (MK2, MK3) and object (cone, plate, ring) as factor s was applied following Tukey HSD post hoc tests. All analyses were performed using a significance criterion of $p < 0.05$.

Example of somatosensory neurons

For comparison with hand-manipulation-related neurons (n= 50), we also recoded the somatosensory neurons. We analyzed neurons with tactile receptive fields (tactile neurons, n= 14) or proprioceptive responsiveness (proprioceptive neurons, n= 10) on the thumb and/or index fingers. These somatosensory neurons exhibited responses during both pre-contact and post-contact periods, similar to non-selective neurons (Fig.S2). Although we recorded conventional tactile

neurons that encode the timing of contact and release with objects (Fig.S2; tactile neuron), some proprioceptive neurons responded to specific objects (e.g., ring) with finger movements prior to contact with the object (Fig.S2; proprioceptive neuron). Population analysis did not show significant differences between the activity of reaching to grasping neurons and that of tactile, proprioceptive neurons in the pre-contact epoch (Fig.S2B). Furthermore, object selectivity was observed in somatosensory neurons (Fig.S2C), and there was no significant distinction between them and hand manipulation-related neurons. The strict distinction of these neurons from neurons related to based solely on the temporal profile of neural activity poses a significant limitation in interpreting the results of the present study.

Fig. S2

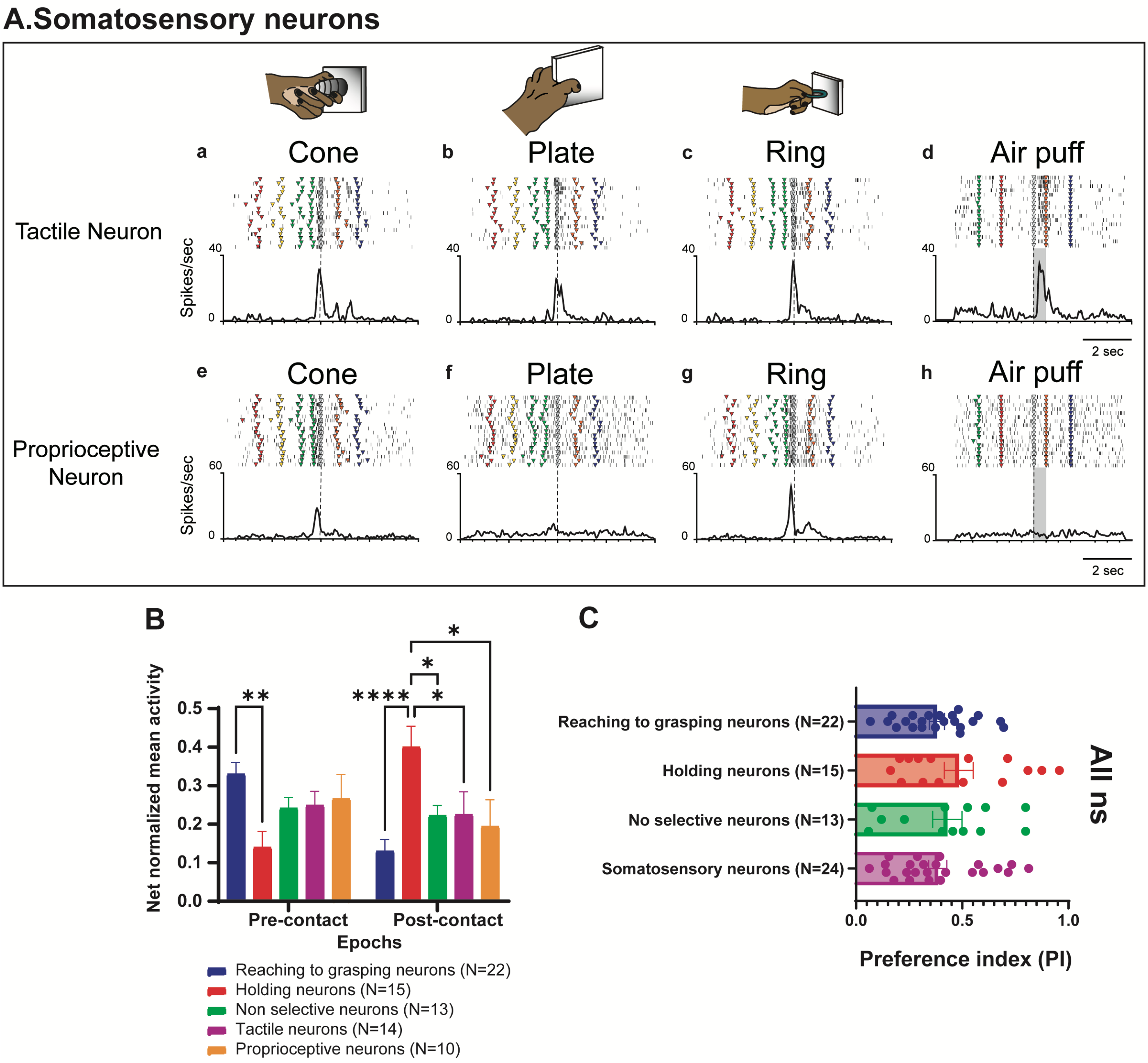

Figure S2: Task-related activity and object selectivity of neurons exhibiting somatosensory receptive fields in D1 (thumb) and D2 (index finger).

Neurons for which somatosensory receptive fields were identified in clinical tests performed prior to the hand manipulation task were classified as somatosensory neurons. The experimenter examined the monkey's hand and, in addition to presenting

stimuli on the skin surface, also carefully assessed the D1 and D2 digits, wrist, elbow, and shoulder joints associated with grasping movements. Of tactile neurons (n= 80) and proprioceptive neurons (n= 41), 14 and 10, respectively, with receptive fields identified in D1 or D2 (skin or joint), were included in the analysis.

A. (Top) An example of a tactile neuron. This neuron showed a superficial tactile receptive field in the tips of D1 and D2. In panel **(a-c)**, this neuron responded to grasping any object. In panel **(d)**, the neuron clearly responds to air puff stimulation. **(Bottom)** An example of a proprioceptive neuron. This neuron responded to passive manipulation of the D2 finger joints. In panels **(e-g)**, this neuron exhibited the strongest response during the grasp of the ring, likely due to its strong association with the motion of the D2 finger joints. In panel (h), the neuron responded to joint movements but not to air puff stimulation on the skin surface of D2.

B. Comparison of the activity of the population of hand-manipulation-related neurons (n= 50) and somatosensory neurons (n= 24).
Mean net normalized response in reaching to grasping neurons (n= 22), holding neurons (n= 15), non-selective neurons (n= 13) and tactile neurons (n= 14), proprioceptive neurons (n= 10) in pre- and post-contact epochs. The bar indicates the standard error of the mean (SEM), with * indicating $p < 0.05$.

C. Preference index for all neuronal types
No significant differences in object selectivity are found for SII/pIC hand-manipulation-related and somatosensory neurons. The bar indicates the standard error of the mean (SEM).
